# Real-world Speech Communication Experiences of Cochlear Implant Users

**DOI:** 10.1097/ONO.0000000000000081

**Published:** 2025-12-17

**Authors:** Bosung N. Titanji, Aaron C. Moberly, Terrin N. Tamati

**Affiliations:** 1 Meharry Medical College, School of Medicine, Nashville, TN; 2 Department of Otolaryngology-Head and Neck Surgery, Vanderbilt University Medical Center, Nashville, TN.

**Keywords:** Cochlear implants, Communication outcomes, Everyday listening environments, Listening in noise, Self, reported communication challenges

## Abstract

**Objective:**

Cochlear implants (CIs) enable adults with hearing loss to regain access to sound. However, many CI users continue to face difficulties in everyday communication, potentially resulting in fewer social interactions. This study aimed to identify CI users’ self-reported hearing difficulties in everyday listening environments.

**Study Design:**

Cross-sectional survey-based study.

**Setting:**

Tertiary care academic medical center; surveys were completed during visit.

**Patients:**

Thirty-seven postlingually deafened adult CI users and 19 age-matched adults with normal hearing (NH) participated.

**Intervention(s):**

Participants completed the Personal Assessment of Communication Abilities survey, rating their communication difficulty in 12 everyday listening environments on a 5-point scale (1 = no difficulty, 5 = very much difficulty).

**Main Outcome Measure(s):**

Self-reported communication difficulty levels in the 12 listening environments with comparisons between CI users and NH peers, and across different environments within each group.

**Results:**

CI users reported significantly more difficulty than NH peers in all 12 environments (*P* < 0.001). Listening context significantly affected difficulty ratings (*P* = 0.003), with a significant group-by-environment interaction (*P* = 0.006). CI users perceived large groups, concerts/movie theaters, and restaurants/cafés most challenging, while one-to-one conversations, outdoors, and conversations in small groups were the least difficult. Outdoor settings and landline phone use showed the greatest differences between groups.

**Conclusions:**

Adult CI users face variable communication challenges in everyday environments. Identifying the everyday listening challenges of adult CI users will help shed light on real-world CI benefits and provide specific targets for rehabilitation.

Approximately 7.6% of adults in the world experience disabling hearing loss (1). Cochlear implants (CIs) are the standard of care for adults living with severe to profound sensorineural hearing loss and ideally allow them to communicate more effectively in everyday situations. Previous research has shown that CIs increase speech perception, which in turn promotes effective communication, social involvement, and mental health (2,3). However, little is known about the real-world experiences of CI users and which listening scenarios are perceived to be more or less challenging.

Although CIs enable adults with hearing loss to regain some hearing function, adult CI users still face challenges. CI users rely on a spectrotemporally degraded signal. The sound signal that is processed by the CI loses frequency and temporal detail, limiting the acoustic detail available to the listener to perceive and understand speech. As a result, CI users have poorer speech recognition abilities compared to normal hearing (NH) peers (4). Further, these limitations likely contribute to the varying levels of hearing and communication difficulty that CI users experience in everyday, real-world environments (4,5). Being able to successfully understand speech and communicate in noisy and complex listening environments requires users to quickly adapt and adjust to the changes in signals and surroundings (6). However, the standard clinical tests commonly used to assess CI outcomes offer only a limited view of speech communication abilities (6). These clinical tests often have oversimplified setups and use sentences that are relatively short, linguistically simple, and carefully read by a single talker (or few talkers) with no discernible regional or foreign accent (6). Although speech-in-noise environments are among the most challenging for listeners, speech-in-noise testing likely fails to capture the dynamic and unpredictable nature of everyday communication, which involves competing talkers, shifting topics, and ever-changing environmental contexts (7). As such, these tests provide baseline insights but do not adequately capture everyday listening scenarios (5,8).

Self-report assessments have been used to gain insight into the difficulties that CI users face in real-world settings. Ecological momentary assessments (EMAs) have been used to analyze social engagement and listening behavior in daily life (9). The experiences of CI users with music, through both intentional listening and passive listening during conversations, have been evaluated using open-ended questionnaires (10). Self-report questionnaires have emerged as means of evaluating other aspects of real-world listening, such as participating in conversations, understanding speech in noisy environments, sound localization, and distinguishing between different instruments and voices/speakers. For example, the Hearing Implant Sound Quality Index questionnaire determines the level of auditory benefit that CI users experience in everyday listening situations (11). These assessments and questionnaires have been found to be easy to use, quick to complete, and relevant to everyday listening situations, allowing clinicians to better understand the persisting hearing difficulties CI users face after implantation.

Some additional listening difficulties have emerged since the introduction of remote communication and face masks amidst the COVID-19 pandemic. Although some of these challenges have shifted postpandemic, the COVID period provided valuable insights into more real-world challenges faced by this population. Daily communication has become more challenging due to the absence of visual cues like lipreading and facial expressions due to face masks (11). Some difficulties CI users have reported include effortful listening, the need to ask for repetition of a message, and feelings of listening-related stress and anxiety (12).

Evaluating the real-world experiences of adult CI users is important for identifying the everyday CI benefits and limitations and for guiding targeted rehabilitation efforts. It is important to note that NH listeners also face real-world challenges in auditory perception and communication. However, for CI users, these challenges are often more pronounced due to the limitations inherent in using a CI. In this study, we aimed to identify CI users’ hearing difficulties in everyday listening environments. The current study used the Personal Assessment of Communication Abilities (PACA) questionnaire to assess the communication difficulties experienced in common real-world listening situations. The questionnaire lists 12 common environments and asks participants to rate the level of hearing difficulty they experience in each. This survey has recently been used to assess communication challenges in adults with hearing loss both before and during the COVID-19 pandemic, a period marked by heightened difficulties due to face masks and social isolation (12,13).

## MATERIALS AND METHODS

### Participants

Thirty-seven postlingually deafened CI users and 19 NH adults were included in this study. Demographic and hearing-related data for the individual CI users can be found in Supplemental Appendix A, https://links.lww.com/ONO/A40. Note that while some participants had missing demographic information, all participants included in the current study completed the PACA questionnaire. All CI users had at least 1 year of experience with their CIs. Among the CI users, there were 22 males and 14 females, with ages ranging from 35 to 83 years (M = 66, SD = 10.6). Eight were unilateral CI users (1 CI only), 17 bimodal CI users (CI + contralateral hearing aid), 11 were bilateral CI users (2 CIs), and 1 participant had an unknown device configuration. Among the NH adults, there were 8 males and 10 females, with ages ranging from 50 to 81 years (M = 68, SD = 9.0). NH was defined as a 4-tone (0.5, 1, 2, and 4 kHz) pure tone average or better than 25 dB HL in the better ear (14). Participants who had previously been enrolled in other research studies at the institution were invited to complete the PACA questionnaire.

### PACA questionnaire

The PACA is a 12-item questionnaire that assesses hearing difficulties across common listening environments (15,16). The environments assessed included one-to-one conversation, conversation in small groups, conversation in large groups, outdoors, concert/movie, place of worship/lectures, watching TV, in a car, telephone—landline, telephone—mobile, and restaurant/cafés. Participants rated their level of communication difficulty on a scale from (1) “no difficulty,” (2) “slight difficulty,” (3) “moderate difficulty,” (4) “quite a lot of difficulty,” (5) “very much difficulty,” and in some cases “not relevant” (n/a). Higher scores on the questionnaire indicate greater difficulty in hearing in real-world environments. Participants also had the option to select “not relevant” if they felt that a particular environment was not relevant to their personal everyday listening experience.

### Data Analysis

We first provide a comprehensive descriptive analysis of participants’ responses to each real-world environment, including means and standard deviations for the Likert scale ratings across all environments. Additionally, we report the proportion of “not relevant” responses for each environment to understand their applicability among CI and NH participants.

Second, a Linear Mixed Model (LMM) was used to assess perceived communication abilities in various real-world environments and between groups. The model was fit using the lmer function within the lme4 package in R (version 4.1.2) (17). The dependent variable was the Likert scale rating for each environment. Fixed effects included the environment, group (CI, NH), and the interaction between environment and group. Participants were included as a random effect to account for repeated measures. The model was fitted using Restricted Maximum Likelihood (REML). Statistical significance was assessed using the Satterthwaite approximation of degrees of freedom for *F*- and *t*-statistics, as implemented in the lmerTest package. All statistical analyses were performed using R (version 4.1.2).

To further examine differences in perceived communication abilities across environments, estimated marginal means were calculated using the emmeans package in R. Pairwise comparisons between environments were conducted with Tukey adjustments to account for multiple comparisons.

Two data points were missing and were assumed to be missing at random. These missing values were excluded from the analysis for the relevant environments, while retaining the rest of the participants’ data in the LMM. Responses marked as “not relevant” by participants were also excluded from the analyses.

## RESULTS

### Descriptive Analysis

The scores from each listening environment for CI users and NH adults are summarized in Table 1. CI users reported more communication difficulty than NH peers in all 12 listening environments. For CI users, conversations in large groups had the highest mean rating of 4.03 (SD = 1.03), indicating the greatest difficulty, while one-to-one conversations had the lowest mean rating of 1.74 (SD = 0.72). For NH, concert/movie had the highest mean rating of 2.37 (SD = 1.16), indicating the greatest difficulty, while Workplace had the lowest mean rating of 1.15 (SD = 0.38). Given the heterogeneity in device configurations among CI users, descriptive PACA scores are reported by device configuration (unilateral, bimodal, and bilateral) in Supplemental Appendix B, https://links.lww.com/ONO/A41. These subgroup data are reported for transparency only, as sample sizes were too small to permit meaningful statistical comparisons.

**Table 1.** Hearing difficulty reported by each group across listening environments

Listening environment	CI Mean ± SD	NH Mean ± SD	No. of CI NR responses (out of 37)	No. of NH NR responses (out of 19)
One-to-one conversation	1.74 ± 0.72	1.25 ± 0.55	0	0
Conversation in small groups	2.50 ± 0.86	1.35 ± 0.49	0	0
Conversation in large groups	4.03 ± 1.03	2.32 ± 0.82	0	1
Outdoors	2.49 ± 0.90	1.75 ± 0.97	0	0
Concert/movie	3.77 ± 1.05	2.37 ± 1.16	2	1
Place of worship/lectures	3.11 ± 1.20	1.41 ± 0.71	0	3
Watching TV	2.55 ± 1.11	1.30 ± 0.57	0	0
In a car	2.61 ± 1.05	1.40 ± 0.50	0	0
Workplace	2.53 ± 0.74	1.15 ± 0.38	23	6
Telephone—Landline	3.37 ± 1.13	1.18 ± 0.39	7	3
Telephone—Mobile	2.79 ± 1.12	1.80 ± 0.62	0	0
Restaurant/cafe	3.75 ± 1.05	2.16 ± 0.83	2	1

NR indicates not relevant.

Both CI users and NH adults reported experiencing the most difficulty in similar environments. Both groups rated conversation in large groups, concerts/movies, and restaurants/cafés as the most difficult listening environments. The scores for each environment were averaged, and the environments were ranked according to their difficulty level (least difficult = 1 and most difficult = 12). Outdoors and telephone—landline showed the greatest ranking difference between CI users and NH adults. For outdoors, it was ranked 2/12 for CI users and 8/12 for NH adults. For Telephone—Landline, it was ranked 9/12 for CI users and 2/12 for NH adults.

The proportion of “not relevant” responses also varied across environments, ranging from 0% in one-to-one conversations to 52% in the workplace, reflecting differences in environment applicability among participants.

### Statistical Analysis

Results demonstrated significant differences in perceived communication abilities across real-world environments for both CI users and NH adults. The LMM analysis revealed significant main effects of environment (F(11, 543.8) = 24.9, *P* < 0.001), indicating that perceived communication ability varied across the environments. Additionally, there was a significant main effect of group (F(1, 54.2) = 51.9, *P* < 0.001), with CI users and NH differing in their overall ratings. A significant interaction of environment × group was also observed (F(11, 543.8) = 4.8, *P* < 0.001), suggesting that the effect of environment on ratings differed between groups. Results of the model are presented in Table 2.

**Table 2.** Linear mixed model (LMM) results

	Sum Sq	Mean Sq	NumDF	DenDF	F value	Pr(>F)
Environment	143.8	13.1	11	543.8	24.9	<0.001
Group	27.2	27.2	1	54.2	51.9	<0.001
Environment × Group	27.5	2.5	11	543.8	4.8	<0.001

Post hoc pairwise comparisons using the Tukey method were conducted to explore these effects. For the main effect of Group, CI users reported significantly greater difficulty (ie, higher ratings) compared with NH adults (t(54) = 7.2, *P* < 0.001). Within the CI group, conversation in large groups was rated significantly higher than: One-to-one conversation, conversation in small groups, place of worship/lectures, watching TV, in a car, workplace, and telephone—landline (*P*’s ≤ 0.014). Restaurant/café was rated significantly more difficult than: One-to-one conversation, conversation in small groups, outdoors, place of worship/lectures, watching TV, workplace, and telephone—landline (*P* ≤ 0.006). Place of worship/lectures was rated significantly higher than one-to-one conversation, conversation in small groups, and outdoors (*P* ≤ 0.007). Telephone—landline was rated significantly higher than one-to-one conversation, conversation in small groups, outdoors, watching TV, in a car, and workplace (*P* ≤ 0.008). Finally, conversation in small groups, outdoors, watching TV, in a car, and telephone—Mobile were also rated significantly higher than one-to-one conversation (*P* ≤ 0.003).

Within the NH group, conversation in large groups was rated significantly higher than: one-to-one conversation, conversation in small groups, outdoors, place of worship/lectures, watching TV, in a car, workplace, telephone—landline, and telephone—Mobile (*P* ≤ 0.020). Concert/movie was also rated significantly higher than: One-to-one conversation, conversation in small groups, outdoors, place of worship/lectures, watching TV, in a car, workplace, and telephone—mobile (*P* ≤ 0.007). Finally, the restaurant/café was rated significantly higher than: One-to-one conversation, watching TV, in a car, workplace, and telephone—landline (*P* ≤ 0.008). Results of the comparisons for each group are presented in Supplemental Appendix C, https://links.lww.com/ONO/A42.

## DISCUSSION

The primary goal of the current study was to evaluate and compare the hearing difficulties experienced by CI users and NH listeners in everyday real-world environments. As expected, our study showed that CI users reported more difficulty in all 12 environments compared with their NH peers. However, it is important to note that the ratings are subjective. For CI users, “no difficulty” may correspond to 70% comprehension, while for NH adults, “no difficulty” may correspond to 100% comprehension. O’Neill et al (5) found that NH listeners considered speech very difficult when they understood only 69%, while CI users only gave the “very difficult” rating when they understood only 14% (5). CI users were more tolerant of drops in speech understanding than NH listeners, often not perceiving their listening experience as more difficult, even when words were unclear, possibly because of their expectations. Although these considerations make it difficult to estimate the degree to which CI users experience challenging environments relative to their NH peers, this finding confirms that CI users continue to experience challenges in everyday communication even with long-term CI use.

Some environments were also perceived as being more difficult than others across both groups. For one-to-one conversations, the overall difficulty level for both CI users and NH adults ranged from no difficulty to slight difficulty. One-to-one conversations were the least challenging for CI users, possibly due to the isolated sound source, the ability to lip-read, and often familiarity with the speaker (5,18). Similar to clinic and laboratory testing, simple one-to-one conversations are likely to occur in a controlled environment. In contrast, conversation in large groups proved to be the most challenging for both CI users and their NH peers, ranking as the most difficult among the 12 listening environments for CI users. This difficulty may stem from the challenges CI users face in large groups, where environments with increased background noise and overlapping voices, such as social gatherings, conferences, and meetings, require the processing of multiple sounds simultaneously (10,19).

Outdoor environments showed the largest difference between CI users and NH peers. Notably, CI users ranked outdoor environments easier than NH adults (2/12 vs 8/12, respectively). This may be due to CI users employing strategies to isolate themselves from noise, while NH listeners may engage in more challenging outdoor environments. The difference in rankings highlights the varying perceptions of CI users and NH adults. While NH adults likely viewed outdoor spaces as crowded and noisy, CI users may have perceived them as less disruptive. Concerts/movies and restaurants/cafés were the other 2 most challenging listening environments for both CI users and their NH peers (20).

Our results are consistent with previous studies regarding CI users’ hearing difficulties in real-world environments (5). In an EMA study by O’Neill et al (5), CI users were asked to rate the difficulty of hearing in their current environment (home, work, leisure environment, in transit, indoors, and outdoors). Poor-performing CI users reported understanding speech in their environment as “somewhat difficult” significantly more of the time compared with their NH peers, while good CI users fell between “no difficulty” and “somewhat difficult” (5). These findings align with our study, where CI users mostly rated hearing difficulty as more than slightly difficult in most environments, while NH listeners reported minimal difficulty. Additionally, CI users reported strategies to adapt to their environment, such as watching mouth movements or facial expressions, positioning themselves closer to the speaker, and piecing together or even pretending to understand what was said (5). In a study of 143 NH adults by Taylor et al (15), the most challenging listening environments were conversation in large groups, conversation in small groups, restaurants/cafes, and the workplace for NH adults. In these 4 environments, NH rated their hearing difficulty as “moderate difficulty” and “slight difficulty” (15). Similarly, our NH participants identified conversations in large groups and restaurants/cafés among the most difficult, along with concerts/movies and telephone—mobile. NH adults rated their hearing difficulty as “slight difficulty” in these 4 environments. These overlapping findings support the validity of our results for NH listeners. In a study by Young et al (13), 12 CI users were surveyed regarding communication abilities using the PACA survey before and during the COVID-19 pandemic. CI users reported “slight” to “moderate” difficulty across 12 environments, with a small increase in difficulty during the pandemic (mean: 2.41 vs 2.78). In contrast, our 37 CI users reported “slight” to “quite a lot” of difficulty across the same environments (mean: 2.94). The greater reported difficulty in our study may reflect differences in sample size, as our larger sample likely included CI users with a broader range of real-world communication abilities.

Our findings suggest that even in situations that we might expect to be easier for CI users, such as one-to-one conversations and conversation in small groups, they still encounter communication difficulties. These difficulties can pose significant challenges, potentially resulting in increased fatigue and potential social withdrawal (21,22). Surprisingly, studies have shown that social isolation during the COVID-19 pandemic decreased, possibly due to spending more time at home, which offers a quieter and more controlled environment compared with workplaces or other social settings (13). This, in turn, resulted in improved speech understanding and reduced listening effort (23). Other studies examining the impact of the COVID-19 pandemic on CI users’ listening difficulties found a negative relationship, with face masks and social distancing reducing speech understanding and causing avoidance of places where masks were required, which led to greater social isolation (12). These studies emphasize the unique challenges CI users face and the varying levels of communication difficulty they experience in everyday listening environments, underscoring the need for personalized rehabilitation.

It is critical to evaluate CI users’ real-world experiences to better understand their functional benefits and limitations and develop targeted rehabilitation strategies. While CIs have been shown to benefit patients with hearing loss, there is still variability in speech and language outcomes after implantation (6). CI users have distinct developmental and hearing backgrounds, resulting in differences in hearing ability (6). Although key demographic, audiologic, and surgical predictors can provide a starting point, there is still considerable variability in patient-reported outcomes that may be more aligned with CI users’ unique experiences and needs. Audiometric test results do not always reflect the challenges an individual faces in real-life situations. The PACA questionnaire highlights aspects of speech communication that are not evaluated in clinical settings. More specifically, the PACA questionnaire allows clinicians to identify specific situations where hearing is difficult and meaningful to an individual CI user. Clinicians can then tailor CI programming and mapping strategies, as well as targeted auditory training, to address these challenges. Thus, collecting this information supports more patient-centered care and helps advance the broader understanding of CI user needs in real-world communication.

However, some limitations should be noted. First, the sample size was relatively small, which limits the generalizability of the results to a broader population. Additionally, due to the small sample size, we were unable to control for several factors known to contribute to outcomes, such as device configuration and age, which could have influenced the perceived difficulty of the various environments. Descriptive PACA scores by device configuration are included in Supplemental Appendix C, https://links.lww.com/ONO/A42. Across configurations, bilateral CI users generally reported lower PACA scores (ie, less difficulty). However, subgroup sizes were too small and unbalanced, so patterns should be interpreted with caution. Next, NH adults were similar in age as CI users, which helped to account for age-related differences to some extent. However, lifestyle factors such as employment status (eg, working vs retired) were not matched between groups. Finally, hearing thresholds were only recorded for the better ear, so we could not confirm that the NH participants did not have any hearing loss in 1 ear. Not matching for age or lifestyle, or having hearing loss in 1 ear, could affect the kinds of communication environments participants regularly encounter and, in turn, influence their reported listening difficulty. Another limitation of the study is the presence of missing data from some participants, which may introduce bias or reduce the representativeness of the sample. However, the amount of missing data was minimal. All participants in this study were experienced CI users, which means their perceptions of hearing difficulty in everyday environments may have been influenced by long-term adaptation to the device. Over time, CI users may become more accustomed to various listening situations, potentially altering their perceived challenges in these environments. As a result, the findings may not reflect the experiences of new CI users.

Additionally, it is unclear the extent to which self-reported communication difficulties align with behavioral speech recognition scores. However, it is also important to note that the PACA captures subjective, self-reported difficulties in everyday listening situations, which do not necessarily align with clinical test results. Ramakers et al (24) explored the relationship between subjective questionnaire responses and objective measures like speech perception and localization in adult CI users. Their study found weak to moderate negative correlations between self-reported speech understanding and objective speech-in-noise performance, and a moderate positive correlation between subjective spatial hearing and measured localization ability (24). This suggests that while clinical tests provide useful information about specific auditory skills, self-report tools like the PACA offer additional insight into how users experience communication challenges in daily life. Future studies should examine the real-world difficulties of new CI users, how perceived difficulties may change over time during adaptation to the CI, and how self-reported difficulties on the PACA relate to speech recognition outcomes.

## CONCLUSION

The current study shows that CI users continue to face auditory perception and communication challenges in real-world listening environments. Even in situations where we might expect CI users to perform relatively well, significant difficulties persist, potentially contributing to issues such as increased fatigue and social withdrawal. By identifying the specific listening challenges experienced by adult CI users, we can better understand the functional benefits of CIs in everyday environments and pinpoint targeted areas for rehabilitation. Future research should further explore the relationship between PACA scores and other real-world factors, such as listening effort, fatigue, and social isolation, to provide a more comprehensive understanding of how CIs impact daily life.

## FUNDING SOURCES

None declared.

## CONFLICT OF INTEREST

A.C.M. serves as CMO and on the Board of Directors for Otologic Technologies and as a paid consultant for Amgen. For the remaining author, none declared.

## DATA AVAILABILITY STATEMENT

De-identified data supporting the findings of this study are available from the corresponding author upon reasonable request. The PACA questionnaire can be obtained from the original developers.

## Supplementary Material


