# Supplementary material for: Real-world Speech Communication Experiences of Cochlear Implant Users
Source: Otol Neurotol Open. 2025 Dec 17;5(4):e081. doi: 10.1097/ONO.0000000000000081 (PMC12737855; doi:10.1097/ONO.0000000000000081)
Supplement: Supplementary file 1 [file ono-5-e081-s001.pdf]

Appendix A. Demographic and hearing-related information of adult CI users

Subject Number	Gender	Age (years)	Age at implantation (years)	Duration of deafness (years)	Configuration	Implant brand	Configuration/ Contralateral hearing aid	CID (% words correct)
1	Female	65	54	24	Bilateral	Cochlear	Bilateral	90
2	Female	55	44	31	Unilateral	Cochlear	Left	60
3	Male	55	50	34	Bilateral	Cochlear	Bilateral	90
4	Male	83	76	33	Bimodal	Cochlear	Right	84
5	Female	35	31	21	Unilateral	Cochlear	Left	76
6	Male	61	55	7	Bimodal	Cochlear	Right	84
7	Male	83	82	33	Bimodal	Cochlear	Left	66
8	Male	69	68	27	Bilateral	Cochlear	Left	82
9	Male	73	73	33	Bimodal	Cochlear	Right	Missing
10	Female	57	48	50	Bimodal	Cochlear	Right	90
11	Female	76	68	31	Unilateral	Cochlear	Left	34
12	Male	75	75	35	Bimodal	Cochlear	Right	Missing
13	Female	54	55	53.5	Bimodal	Cochlear	Right	Missing
14	Female	49	N/A	49	Bimodal	Cochlear	Right	Missing
15	Male	63	63	25	Bimodal	Cochlear	Right	Missing
16	Male	79	76	19	Bimodal	Cochlear	Right	70
17	Female	69	56	54	Bilateral	Cochlear	Bilateral	84
18	Female	68	59	58	Unilateral	Cochlear	Right	70
19	Male	79	74	76	Bimodal	Cochlear	Left	56
20	Male	59	57	4	Bilateral	Cochlear	Bilateral	92
21	Male	78	72	21	Bilateral	Cochlear	Bilateral	76
22	Male	76	74	53	Unilateral	Cochlear	Left	18
23	Female	67	58	31	Bilateral	Cochlear	Bilateral	90
24	Male	66	60	52	Bimodal	Cochlear	Left	92
25	Female	64	61	11	Bimodal	Cochlear	Right	92
26	Male	56	Missing	Missing	Bilateral	Missing	Missing	66
27	Female	65	43	65	Unilateral	Bionics	Left	46
28	Female	68	Missing	Missing	Bilateral	Missing	Missing	80
29	Female	55	46	55	Unilateral	Cochlear	Bilateral	Missing
30	Missing	Missing	Missing	Missing	Missing	Missing	Missing	Missing
31	Male	82	82	Missing	Bimodal	Cochlear	Right	Missing
32	Male	68	68	Missing	Bimodal	Cochlear	Right	Missing
33	Male	67	67	22	Bimodal	Cochlear	Right	Missing
34	Male	65	65	15	Unilateral	Cochlear	Left	Missing
35	Male	65	65	30	Bimodal	Missing	Right	Missing

36	Male	74	Missing	Missing	Bilateral	Missing	Missing	Missing
37	Male	53	36	50	Bilateral	Cochlear	Bilateral	56

Note: CID indicates Central Institute for the Deaf (CID) Auditory Test W-22 (Hirsh et al., 1952)
