# Supplementary material for: Real-world Speech Communication Experiences of Cochlear Implant Users
Source: Otol Neurotol Open. 2025 Dec 17;5(4):e081. doi: 10.1097/ONO.0000000000000081 (PMC12737855; doi:10.1097/ONO.0000000000000081)
Supplement: Supplementary file 2 [file ono-5-e081-s002.pdf]

Appendix B. Hearing difficulty reported by device configuration (unilateral, bimodal, unilateral) across listening environments

**Listening environment**	**Bilateral mean ± SD n=11**	**Bimodal mean ± SD n=17**	**Unilateral mean ± SD n=8**
One-to-one conversation	1.55 ± 0.69	1.88 ± 0.70	1.75 ± 0.89
Conversation in small groups	2.36 ± 0.67	2.71 ± 1.10	2.38 ± 0.52
Conversation in large groups	3.45 ± 1.37	4.41 ± 0.62	3.88 ± 0.99
Outdoors	2.09 ± 0.83	2.63 ± 0.72	2.38 ± 0.92
Concert/movie	3.64 ± 1.21	3.86 ± 1.03	3.75 ± 1.04
Place of worship/lectures	2.45 ± 1.04	3.35 ± 1.17	3.38 ± 1.41
Watching TV	2.27 ± 1.01	2.71 ± 1.05	2.38 ± 1.19
In a car	1.91 ± 0.70	3.12 ± 1.05	2.50 ± 1.07
Workplace	2.50 ± 1.29	2.40 ± 0.55	2.75 ± 0.50
Telephone - Landline	3.22 ± 1.30	3.58 ± 1.08	3.13 ± 1.13
Telephone - Mobile	2.27 ± 0.90	3.24 ± 1.20	2.63 ± 1.06
Restaurant/Cafe	3.00 ± 0.94	4.25 ± 0.86	3.75 ± 1.16
Total	2.53 ± 0.68	3.19 ± 0.60	2.88 ± 0.75
