# Supplementary material for: Real-world Speech Communication Experiences of Cochlear Implant Users
Source: Otol Neurotol Open. 2025 Dec 17;5(4):e081. doi: 10.1097/ONO.0000000000000081 (PMC12737855; doi:10.1097/ONO.0000000000000081)
Supplement: Supplementary file 3 [file ono-5-e081-s003.pdf]

Appendix C. Appendix B shows pairwise comparisons of perceived communication abilities across environments, based on estimated marginal means (EMMs) that adjust for other variables in the model. Tukey-adjusted tests were used to assess whether differences between each pair of environments were statistically significant. The number value is the difference in EMMs. A positive value means the first environment had a higher perceived communication score than the second.

1. Pairwise Comparisons of Perceived Communication Abilities Across Environments of Adult CI Users

	One to one conversation	Conversation in small groups	Conversation in large groups	Outdoors	Concert/movie	Place of worship/ lectures	Watching TV	In a car	Workplace	Telephone-Landline	Telephone-Mobile	Restaurant/ Cafe
One-to-one conversation		-0.76***	-2.24***	-0.69**	-2.03	-1.35***	-0.81***	-0.86***	-0.70 (ns)	-1.61***	-1.05***	-2.01***
Conversation in small groups			-1.49***	0.06 (ns)	-1.27***	-0.59*	-0.05 (ns)	-0.11 (ns)	0.06 (ns)	0.85***	0.30 (ns)	1.25***
Conversation in large groups				1.55***	0.22 (ns)	0.89***	1.43***	1.38***	1.55***	-0.64*	-1.19***	-0.24 (ns)
Outdoors					-1.33***	-0.66**	-0.12 (ns)	-0.17 (ns)	0.00 (ns)	0.91***	0.36 (ns)	1.31***
Concert/movie						0.67**	1.21***	1.16***	1.33***	-0.42 (ns)	-0.97***	-0.02 (ns)
Place of worship/lectures							0.54 (ns)	0.49 (ns)	0.66 (ns)	0.25 (ns)	-0.30 (ns)	0.66**
Watching TV								-0.05 (ns)	0.11 (ns)	0.80***	0.24 (ns)	1.20***
In a car									0.17 (ns)	0.74**	0.19 (ns)	1.14***
Workplace										0.91**	0.36 (ns)	1.31***
Telephone - Landline											0.55 (ns)	-0.40 (ns)
Telephone - Mobile												-0.95***
Restaurant/Cafe												

2. Pairwise Comparisons of Perceived Communication Abilities Across Environments of Normal Hearing Peers

	One to one conversation	Conversation in small groups	Conversation in large groups	Outdoors	Concert/movie	Place of worship/ lectures	Watching TV	In a car	Workplace	Telephone - Landline	Telephone-Mobile	Restaurant/ Cafe
One-to-one conversation		-0.11 (ns)	-1.11***	-0.53 (ns)	-1.16***	-0.19 (ns)	-0.05 (ns)	-0.16 (ns)	0.09 (ns)	0.12 (ns)	-0.58 (ns)	-0.88*
Conversation in small groups			-1.00**	-0.42 (ns)	-1.06***	-0.09 (ns)	0.05 (ns)	-0.05 (ns)	0.20 (ns)	-0.22 (ns)	0.47 (ns)	0.78*
Conversation in large groups				0.58 (ns)	-0.06 (ns)	0.91*	1.05***	0.95**	1.20***	-1.22***	-0.53 (ns)	-0.22 (ns)
Outdoors					-0.63 (ns)	0.33 (ns)	0.47 (ns)	0.37 (ns)	0.62 (ns)	-0.65 (ns)	0.05 (ns)	0.36 (ns)
Concert/movie						0.97**	1.11***	1.00**	1.25***	-1.28***	-0.58 (ns)	-0.28 (ns)
Place of worship/lectures							0.14 (ns)	0.04 (ns)	0.29 (ns)	-0.31 (ns)	0.39 (ns)	0.69 (ns)
Watching TV								-0.11 (ns)	0.14 (ns)	-0.17 (ns)	0.53 (ns)	0.83*
In a car									0.25 (ns)	-0.28 (ns)	0.42 (ns)	0.73 (ns)
Workplace										-0.03 (ns)	0.67 (ns)	0.97*

Telephone - Landline											-0.70 (ns)	-1.00**
Telephone - Mobile												-0.30 (ns)
Restaurant/Cafe												

Note:
* $p<.05$
** $p<.01$
*** $p <.001$
(ns) = not significant
Positive values indicate greater difficulty in the environment listed in the row; negative values reflect greater difficulty in environment listed in the column.
